# Specific circulating neutrophils subsets are present in clinically stable adults with cystic fibrosis and are further modulated by pulmonary exacerbations

**DOI:** 10.3389/fimmu.2022.1012310

**Published:** 2022-09-29

**Authors:** Clémence Martin, Théo Dhôte, Maha Zohra Ladjemi, Muriel Andrieu, Souganya Many, Vaarany Karunanithy, Frédéric Pène, Jennifer Da Silva, Pierre-Régis Burgel, Véronique Witko-Sarsat

**Affiliations:** ^1^ Institut Cochin, Inserm U1016, Centre National de la Recherche Scientifique (CNRS) UMR8104, Université Paris-Cité, Paris, France; ^2^ Service de Pneumologie & Centre de Référence Maladies Rares Mucoviscidose, site coordonnateur, Hôpital Cochin, Assistance Publique-Hôpitaux de Paris-Centre & Université de Paris-Cité, Paris, France; ^3^ Service de Médecine intensive & Réanimation, Hôpital Cochin, Assistance Publique-Hôpitaux de Paris-Centre & Université de Paris-Cité, Paris, France

**Keywords:** neutrophils, cystic fibrosis, low density neutrophils, subpopulation, myeloid-derived suppressor cell (MDSC)

## Abstract

The progressive lung destruction in cystic fibrosis (CF) is tightly associated with chronic bacterial infection and neutrophil-dominated airway inflammation. CF pulmonary disease is complicated by episodes of acute exacerbations, contributing to irreversible lung damage. We hypothesized that circulating subsets of neutrophils from clinically stable adults with CF present some phenotypic specificities that could amplify their activation during an infectious episode. The aim of the present study was to examine the different neutrophil subsets in whole blood and in the low density neutrophils (LDN) that co-purify with peripheral blood mononuclear cells (PBMC) in clinically stable adults with CF and in CF adults during pulmonary exacerbations compared to healthy donors. Blood samples were obtained from 22 adults with CF (16 in stable state and 6 during pulmonary exacerbations) and from 20 healthy donors. Flow cytometry analysis of 13 different markers related to lineage (CD45, CD15), maturity (CD16, CD10, and CD33), activation (CD62L, CD11b, CD66b, and CD114), metabolism (GLUT-1, LOX1) and immunosuppression (PD1, PD-L1) was carried out within whole blood and within the LDN fraction. Unsupervised analysis of flow cytometry data was performed using visual t-distributed stochastic neighbor embedding (vi-tSNE). A significant increase in the CD11b expression in neutrophils from CF patients during exacerbations was observed compared to neutrophils from stable CF patients or to healthy donors, indicative of a circulating activation state due to an infectious status. The percentage of LDN was not increased in stable CF patients but increased during exacerbations. Analysis of neutrophil subsets using the double CD16/CD62L labeling revealed a significant increase in the CD16^high^/CD62L^low^ subset in all CF patients compared to healthy donors. In contrast, an increase in the CD16^low^/CD62L^high^ subset was observed only in CF patients during exacerbations. Unsupervised analysis identified a PD-L1^high^/CD114^high^ population that was present in stable CF patients and as well as in CF patients during exacerbations.

## Introduction

Neutrophils are key cells of innate immunity since they represent the vast majority of circulating leukocytes. More recently, their immunomodulatory role has been recognized since neutrophils have the ability to dialog with other cells involved in innate and adaptive immunity (1, 2). Upon an infectious challenge, neutrophils are recruited to the site of the injury. During their migration their effector mechanisms are safely constrained to prevent tissue damage. Neutrophils can release their powerful mediators such as NADPH oxidase-dependent reactive oxygen species (ROS), antibiotic proteins and proteases only after the phagocytosis of the bacteria within the infected tissue (3, 4). An appropriate coordinated response results both in the clearance of the infection and in the resolution of the inflammation (5). Neutrophil apoptosis plays a key role in the restoration of tissue homeostasis (6, 7). This process is unfortunately disrupted and neutrophil accumulation in airways is a hallmark of the pathophysiology of cystic fibrosis (CF) characterized by a defect in the resolution of inflammation (8).

For a long time confined to a homogeneous population, we now know that there are different subsets of neutrophils that differ in maturity, density, and inflammatory properties. First, low density neutrophils (LDN), which were first described in auto-immune diseases (9), cancer and sepsis (10). Then, granulocytic-myeloid derived suppressor cells (G-MDSC), were described in cancer (11). They are heterogeneous immature populations, endowed with negative regulation of the innate and adaptive response, in particular with T-suppressive capacities. There is convergent evidence that lectin-type oxidized LDL receptor-1 (LOX-1) and Programmed Death-Ligand 1 (PD-L1) are two surface receptors overexpressed by neutrophils showing immunosuppressive abilities in particular T-cell suppression (12, 13)

CF is characterized by respiratory impairment, with bronchiectasis, chronic airway bacterial infection, and repeated episodes of acute pulmonary exacerbations (14). Airway inflammation that occurs during chronic bacterial infection and acute exacerbations is considered responsible for progressive destruction of the airways and lung parenchyma, sometimes leading to respiratory failure. Definition of pulmonary exacerbations in CF is a source of controversy and developing a reliable biomarker could help in patient monitoring (15).

Neutrophils and its proteases have been identified in the airway of infants with CF and was associated with subsequent development of bronchial dilatation at school age (16, 17). Mucus plugs are found within airways and are mainly composed of mucins and neutrophils (18). Although a general defect in innate immunity is now mooted in neutrophils, the question remains: Why don’t neutrophils do a better job ? (19) The neutrophil is the most efficient cell that is equipped to fight bacteria. However, the neutrophil is present in high numbers in CF airways but is ineffective to suppress bacterial infection (20, 21). Several studies have reported disturbance in the bactericidal mechanisms (22–24), in particular related to myeloperoxidasedefect (25).

Little is known about the phenotype of circulating neutrophils in CF. The absolute neutrophil count (26) as well as the neutrophil to lymphocyte ratio (NLR) are increased in CF patients and correlate with disease severity (27). The discovery of different neutrophil subpopulations has introduced a new level of understanding of the defective role of neutrophils in disease (28). Nonetheless, neutrophil heterogeneity has been observed in cancer or autoimmune diseases in the absence of infectious challenge, suggesting that other immune-mediated mechanisms could be involved (29, 30). Whether the chronic inflammatory state, between repeated pulmonary exacerbation episodes that characterize the pathophysiology of CF, can induce either preset subpopulations of neutrophils or simply neutrophils with various activation states is a key issue (31). Indeed, it has been well demonstrated that exposing healthy volunteers to *E. coli* lipopolysaccharide induces different neutrophil subpopulations that has been characterized by their CD16/CD62L differential membrane expression, their nuclear morphology and their phagocytosing capacities (32, 33). For example, one neutrophil subpopulation had a lower CD16 surface expression, with a banded morphology and increased phagocytosis capacities (34) whereas the other had a lower CD62L surface expression, with a hypersegmented morphology, and had T-suppressive capacities (35). This seminal observation clearly established that neutrophil heterogeneity is a consequence of a pathogen-mediated activation which has been also evidenced in severe inflammation such as sepsis (13) and trauma (36).

In the present study we hypothesized that circulating subsets of neutrophils from stable CF patients present phenotypic specificities that could amplify their activation during an infectious episode. The aim of this study was to describe the phenotype of blood neutrophils in CF and to characterize specific subpopulations, including LDN.

## Materials and methods

### Patient recruitment and characteristics

Patients were recruited from the Cystic Fibrosis Reference Centre, in the Pulmonology Department of Cochin Hospital in Paris, France. This study was approved by the hospital Ethic committee (Committee for the Protection of Persons number: 19005 –2137-18.11.26. 50423). Each patient provided written informed consent. The inclusion period spanned from April 04, 2019 to March 11, 2020. Twenty-two patients with CF were included and 20 healthy donors were recruited from the French Blood Bank (Etablissement Français du sang). The main characteristics of the CF population are described in **Table 1**. The samples were classified in three groups: Healthy donors (N = 20), Stable CF patients (N = 16), CF patients on exacerbation (N = 6). There is no definitive consensus on the definition of acute exacerbation; in the present study, an exacerbation was defined as a worsening of symptoms and/or lung function leading to the introduction of systemic antibiotic therapy (15).

**Table 1:** Demographic and clinical characteristics of patients with cystic fibrosis.

CF patients (n = 22)	
Age (y), mean (SD)	28.2 (9.9)
Sex, n (%) Male	17 (77.3)
CFTR Genotype, n (%) Class I-III mutations Class IV-V mutations	17 (77.3) 5 (22.7)
Sweat chloride concentration, median (interquartile range)	105 (70-110)
Respiratory status, Male/Female (%) Stable	13/3 (72.7)
Exacerbation	4/2 (27.3)
ppFEV1, median (interquartile range)	75 (46.5-98.5)
Sputum bacteria, n (%) P. aeruginosa M. abscessus Allergic bronchopulmonary aspergillosis	11 (50.0) 3 (13.6) 8 (36.4)
Medication, n (%) Azithromycin Oral corticosteroid Anti-IL5 Ab	11 (50.0) 4 (18.2) 8 (36.4)
CFTR modulator, n (%) Ivacaftor Lumacator-Ivacaftor Elexacaftor-Tezacaftor-Ivacaftor	5 (22.7) 1 (4.5) 2 (9.1)
IV antibiotic course in the last year, median (interquartile range)	1 (0-2.5)
IV antibiotic days in the last year, median (interquartile range)	14 (0-24.5)

ppFEV1, percent-predicted Forced Expiratory Volume in 1 second.

### Blood processing and cell isolation

Blood was collected into Vacutainer EDTA K2 (1.8 mg/mL, Becton Dickinson, Franklin Lakes, US) from CF patients and healthy donors and samples were processed within 4 hours of collection. Normal density neutrophils (NDN) and peripheral blood mononuclear cells (PBMC) were isolated from blood by ficoll gradient as previously described (26). Briefly, 12 mL of whole blood was layered onto 15 mL Ficoll-Histopaque (Density 1.077 g/mL, Sigma) and centrifuged at 700 g for 30 min. PBMC were reserved. The red blood cells and neutrophils were resuspended in 10 mL PBS (phosphate-buffered saline, ThermoFisher 10010023, Waltham, MA USA) and 15 mL Dextran 2% (Sigma 31392, diluted in NaCl 0.9%). Sedimentation time was 50 minutes. The supernatant containing the neutrophils was collected and centrifuged at 500 g for 5 minutes. Next, the supernatant was discarded, and the remaining red blood cells were lysed in 10 mL NaCl 0.2% for 40 sec, then 1 mL of NaCl 1.6% was added and PBS was filled until 50 mL. The sample was centrifuged for 5 min at 500 g and the procedure of lysis was repeated for another twice. The supernatant was discarded and the pellet containing the NDN was resuspended in RPMI complete plus 10% FBS (RPMI complete: RPMI 1640 GlutaMAX (ThermoFischer, 61870036) 1% penicillin-streptomycin (Gibco 15140-122) and 1% Hepes Buffer Solution (Gibco, 156630-056)). Isolated neutrophils and PBMC were diluted in Turk`s solution, counted on a hemocytometer KOVA Glasstic Slide (ThermoFisher) and were adjusted at 10 (6) cells/ml for further immunolabelling. It should be noted that the isolation process may cause activation of the neutrophil and thus alter its phenotype

### Neutrophil immune-labelling and flow cytometry analysis

**Figure 1:**
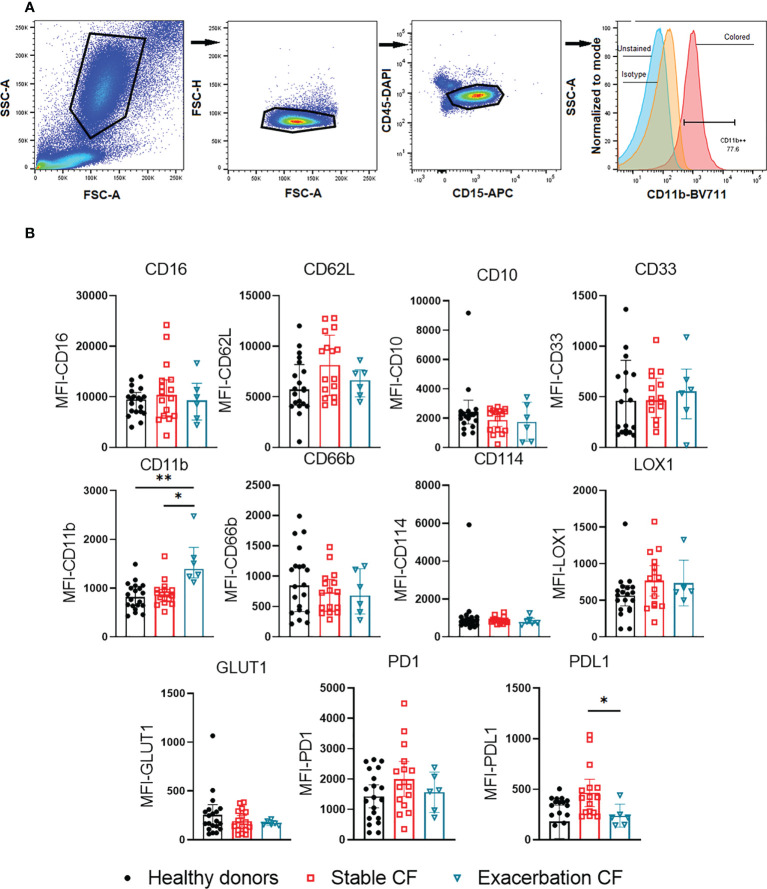
Flow cytometry analysis of whole blood neutrophil phenotype. **(A)** Gating strategy. Granulocytes were gated by FSCxSSC, singlets were identified by FSC-AxFSC-H. CD45+/CD15+ cells were selected, corresponding to neutrophils population. Residual eosinophils, identified as CD16- and CD62L+ cells, were excluded. The use of isotypes allowed to define the positivity of the different gates, the example of CD11b expression is given. **(B)** Surface markers expression was compared within neutrophils from healthy donors (n = 20), stable CF patients (n=16) and CF patients during exacerbation (n=6). All results are presented as median fluorescence intensity (MFI) with interquartile range (Kruskal-Wallis test with Dunn’s multiple comparison test, *p < 0.05, **p < 0.01).

The phenotypic characterization of neutrophil subpopulations was performed in whole blood, in PBMC and in isolated neutrophils by multicolor flow cytometry of 13 membrane markers (**Table 2**). First, the antibody mix was prepared, with one tube containing the 13 specific antibodies and the other tube containing the corresponding labeled isotypes. The antibody or isotype mix were added to the cells and incubated for 10 min on ice in the dark. The whole blood cells were then lysed with Lysing buffer 1X (Becton Dickinson 555899) and washed with PBS. To fix the cells, 200 µL of 1% paraformaldehyde was added to each tube. Finally, 300 µL of Stain Buffer (Becton Dickinson, Franklin Lakes, US) was added for storage at +4°C until acquisition within 24 hours of labeling, using a flow cytometer (LSR Fortessa, Becton Dickinson). The gating strategy was as follows: granulocytes were first selected by size (FSC) and granularity (SSC) analysis. After exclusion of doublets (FSC-H/FSC-A), double labelling with CD45/CD15 resulted in the neutrophil population (**Figure 1A**). Residual eosinophils, identified as CD62L^high^/CD16^neg^, were excluded from the analysis. We used CD16 (FcγRIII), CD10 (neutral endopeptidase) and CD33 (Siglec-3) as maturity markers; CD62L (L-selectin), CD11b (Integrin alpha M), CD66b (CEACAM-8, carcinoembryonic antigen-related cell adhesion molecule 8) and CD114 (G-SCF granulocyte colony stimulating factor receptor) as activation markers; LOX-1 (Lectin-like oxidized low-density lipoprotein receptor-1) and GLUT1 (Glucose transporter 1) as metabolic activity markers and PD-1 (Programmed Death receptor 1) and PD-L1 (Programmed Death Ligand 1) as immune modulation markers. Data analysis was performed using FlowJo v10.8.1 software (BD, Ashland, OR, USA). Expression of each individual surface marker was expressed by the median fluorescence intensity (MFI). In order to identify and visualize neutrophil subpopulations, an unsupervised analysis was performed using the dimensional reduction algorithm visual t-Distributed Stochastic Neighbor Embedding (t-viSNE). This analysis was carried out using the CytoBank platform (Beckman Coulter Life Sciences, Brea, US). A sample of 10.000 CD45+/CD15+ neutrophils was randomly taken from each patient. All cells (n=420.000) were then grouped and subjected to the algorithm to form clusters according to the expression levels of the surface phenotyping markers.

**Table 2:** Antibodies for flow cytometry panel.

	Marker	Fluorochrome	Provider	Clone
**Marked**	CD16	AlexaFluor-700	Biolegend	368
	CD10	PE-Cy-7	Biolegend	HI110a
	CD66b	PerCP-Cy5.5	Biolegend	G10F5
	CD62L	BV-785	Biolegend	DREG-56
	CD11b	BV-711	Biolegend	ICRF44
	CD114	BV-510	Becton Dickinson	LMM741
	PD1	APC-Cy-7	Biolegend	EH12.2H7
	CD33	BV-650	Biolegend	WM53
	CD15	APC	Biolegend	HI98
	LOX-1	PE	Biolegend	15C4
	PD-L1	BV-421	Biolegend	MIH3
	GLUT1	FITC	R&D systems	FAB1418G
	CD45	DAPI	Becton Dickinson	H130
**Isotype**	Mouse-IgG1,k	AlexaFluor-700	Biolegend	MOPC 21
	Mouse-IgG1,k	PE-Cy-7	Biolegend	MOPC 21
	Mouse IgM,k	PerCP-Cy5.5	Biolegend	MM-30
	Mouse-IgG1,k	BV-785	Biolegend	MOPC 21
	Mouse-IgG1,k	BV-711	Biolegend	MOPC 21
	Mouse-IgG1,k	BV-510	Becton Dickinson	X40
	Mouse-IgG1,k	APC-Cy-7	Biolegend	MOPC 21
	Mouse-IgG1,k	BV-650	Biolegend	MOPC 21
	Mouse IgM,k	APC	Biolegend	MM-30
	Mouse IgG2a,k	PE	Biolegend	MM-30
	Mouse-IgG1,k	BV-421	Biolegend	MOPC-173
	Mouse IgG2b,k	FITC	Biolegend	MPC-11
	Mouse IgG2a,k	DAPI	Becton Dickinson	G155-178

### Statistical analysis

Statistical analyses and graphs were performed with Prism version 9 (GraphPad Software, San Diego, US). To compare two populations, a Mann Whitney test was used for unpaired data or a Wilcoxon test for paired data. A Kruskal-Wallis test with Dunn’s post-hoc analysis was performed to compare more than two groups. Differences were considered significant when p < 0.05.

## Results

### Increased CD11b expression on neutrophils from CF patients during exacerbations

Flow cytometry supervised analysis showed no significant difference in the expression of maturity markers (CD16, CD10, CD33), activation markers (CD11b, CD66b, and CD62L), metabolic markers (LOX1, GLUT1) nor immunosuppression and/or exhaustion markers (PD1, PD-L1) between whole blood neutrophils from healthy donors and stable CF patients (**Figure 1B**). In contrast neutrophils obtained from CF patients during exacerbations exhibited a significant increase in CD11b expression as compared to healthy donors and stable CF patients suggesting a higher activation state of neutrophils during exacerbation. We found that PD-L1 expression decreased during exacerbations as compared to stable CF patients.

### Increased percentage of low density neutrophils in CF patients during exacerbations

**Figure 2:**
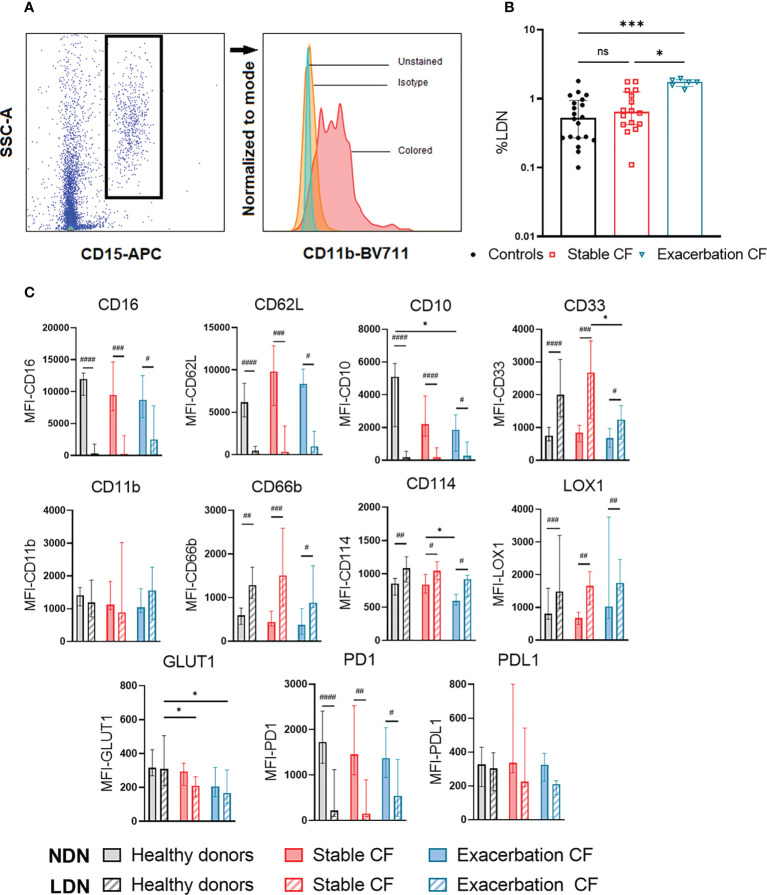
Analysis of the low-density neutrophils (LDN) and normal density neutrophils (NDN). LDN and NDN were isolated using Ficoll gradient. NDN were analyzed within the PBMC ring and NDN were isolated from the pellet **(A)** gating strategy of LDN defined as CD15+ cells within PBMC. **(B)** Proportion of LDN in healthy donors (n=20), stable CF (n = 16) and CF patients during exacerbation (n=6). Data are presented as % of LDN within PBMCs with bars indicating median ± interquartile range (Kruskal-Wallis test with Dunn’s multiple comparison test, *p < 0.05, *** p<0.001). **(C)** Phenotypic characterization of NDN (solid bars) and LDN (hatched bars). Data are expressed as median fluorescence intensity (MFI) for each marker ± interquartile range (Wilcoxon test for intra-group NDN and LDN comparison, #p < 0.05, ##p < 0.01, ###p < 0.001, ####p < 0.0001; Kruskal-Wallis test with Dunn’s multiple tests for inter-group NDN and LDN comparison, *p < 0.05).

LDN were defined as CD15+ cells within PBMC (**Figure 2A**). We observed that the proportion of LDN in CF stable patients was not different from those of the healthy donors (0.80 ± 0.51% vs 0.70 ± 0.45%). In contrast, in the CF exacerbation group, the proportion of LDN was significantly increased as compared to healthy donors (1.71 ± 0.22% vs 0.70 ± 0.45%, p=0.0019) and to stable CF (1.71 ± 0.22%vs 0.80 ± 0.51%, p=0.0086) (**Figure 2B**). The phenotype of isolated NDN and LDN was compared marker by marker (**Figure 2C**). Compared to NDN, LDNs from all groups showed an immature phenotype (CD16 ^low^, CD10^low^, CD33^high^), a high activation state (CD66b^high^, CD62L^low^, CD114^high^) and an increased LOX1 expression. LDN from CF patients (stable CF and during exacerbation) displayed a significant lower GLUT1 expression as compared to those from healthy donors. Moreover, LDN from CF patients during exacerbation showed a lower CD33 expression compared to stable CF patients (**Figure 2C**). This last result must be taken with caution, as the proportions of LDN described here remain low.

NDN from CF patients during exacerbation displayed a significant decrease in CD10 expression compared to healthy donors and in CD114 expression compared to stable CF patients. However, it should be noted that the proportion of CD10 positive NDN was similar (median) in healthy controls (92%), stable CF patients (91.8%) and during exacerbation (86.2%), which is consistent with whole blood analysis.

### Increased CD16high/CD62Llow neutrophil subset in stable CF patients and during exacerbations

**Figure 3:**
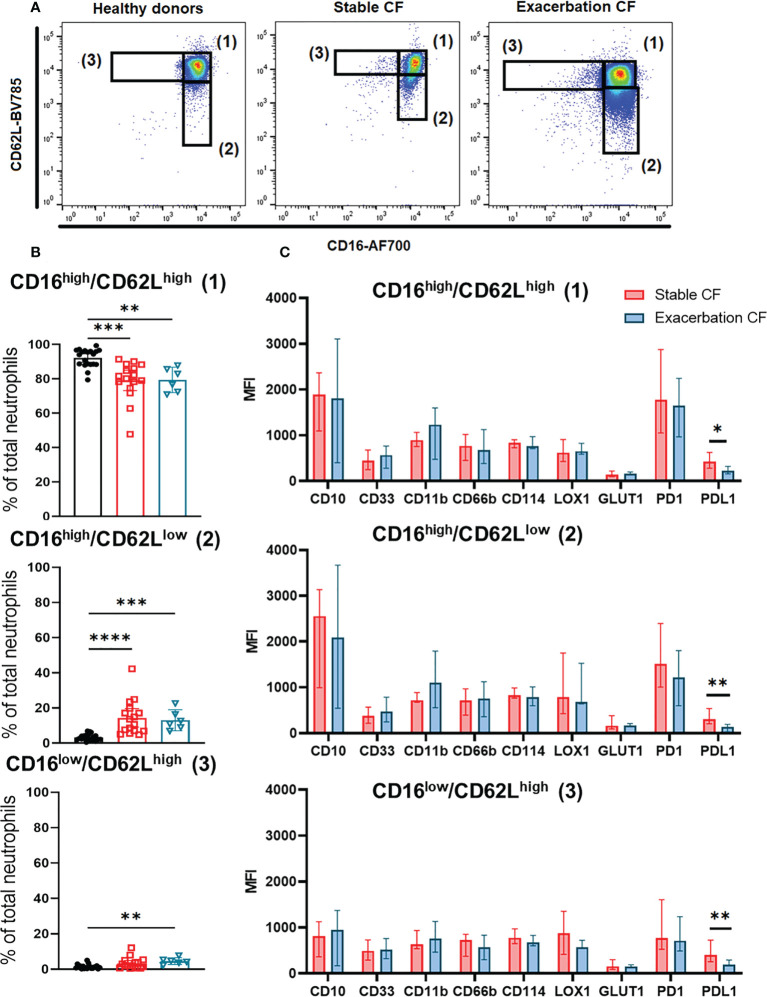
Neutrophils subsets identification defined by CD16 and CD62L expression. **(A)** Representative dot plots of CD16 and CD62L double staining in neutrophils from healthy donors (left panel) and stable CF patients (middle panel) or CF during exacerbation (right panel), defining three distinct subsets: CD16high/CD62Lhigh (1), CD16high/CD62Llow (2) and CD16low/CD62Lhigh (3). **(B)** Distribution of the three CD16/CD62L gated subsets within the healthy donors (n=20), stable cystic fibrosis patients (n = 16) and CF patients during exacerbation (n = 6). Data are presented as % of neutrophils subsets, with bars indicating median ± interquartile range (Kruskal-Wallis test with Dunn’s multiple comparison test, **p < 0.01, ***p < 0.001, ****p < 0.0001). **(C)** Phenotypic characterization of the three neutrophils subsets in stable and CF patients during exacerbation. Results are presented as median fluorescence intensity (MFI) ± interquartile range (Mann-Whitney U test, *p < 0.05, **p < 0.01).

As previously described in other diseases (35, 36), we identified three distinct whole blood neutrophil subsets as referred as CD16^high^/CD62L^high^ (1), CD16^high^/CD62L^low^ (2) and CD16^low^/CD62L^high^ (3) according to the dual CD16/CD62L labeling (**Figure 3A**).

Analysis of this distribution showed an increase in the CD16^high^/CD62L^low^ (2) subpopulation in stable and exacerbation CF patients as compared to healthy donors (14.34 ± 9.93% vs 3.32 ± 1.85%, p<0.0001 and 22.98 ± 26.84% vs 3.32 ± 1.85%, p=0.0002 respectively). The proportion of the CD16^low^/CD62L^high^ (3) subpopulation was increased only in CF patients during exacerbation (4.69 ± 1.69% vs 1.42 ± 1.21%, p=0.0012) (**Figure 3B**).

We observed a significant decrease in PD-L1 expression in the three neutrophil subsets from CF patients during exacerbation compared to those from stable CF patients, whereas CD11b expression tended to increase (**Figure 3C**). This was consistent with data obtained from whole blood neutrophil analysis (**Figure 1B**).

**Figure 4:**
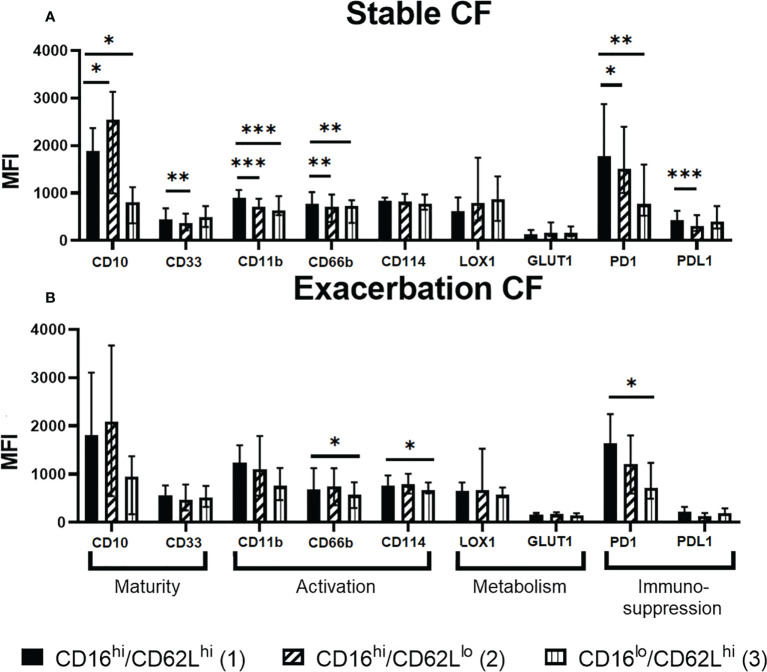
Phenotypic characterization of the CD16/CD62L neutrophil subsets in **(A)** stable CF (n=16) and **(B)** CF patients during exacerbation (n=6). The CD16high/CD62Llow (2) and CD16low/CD62Lhigh (3) subsets were compared to the “classical” CD16high/CD62Lhigh (1) subset by measuring the expression of every other marker. Data are presented as median fluorescence intensity (MFI) ± interquartile range (Wilcoxon test, *p < 0.05 **p < 0.01 ***p < 0.001).

To further characterize these neutrophil subsets, we performed a differential analysis of their phenotype according to the surface markers expression (**Figure 4**). As compared to the “classical” CD16^high^/CD62L^high^ (1) subset, the CD16^high^/CD62L^low^ (2) subset from stable CF group was more mature (increased CD10 and decreased CD33), less activated (decreased CD11b and CD66b) and showed an altered immunomodulation markers expression (increased PD1 and decreased PD-L1). The CD16^low^/CD62L^high^ (3) subset was less mature (decreased CD10), less activated (decreased CD11b and CD66b) and showed a decreased PD1 expression (**Figure 4A**).

We observed a similar phenotype within CD16^high^/CD62L^low^ (2) and the CD16^low^/CD62L^high^ (3) subsets in the exacerbation CF group as compared to the “classical” CD16^high^/CD62L^high^ (1) subset even though all parameters did not reach significance (**Figure 4B**). Of note, CD114 expression was significantly decreased in CD16^low^/CD62L^high^ (3) subset only during exacerbation.

### Identification of a PD-L1high/CD114high neutrophil subset in stable CF patients and during exacerbation

**Figure 5:**
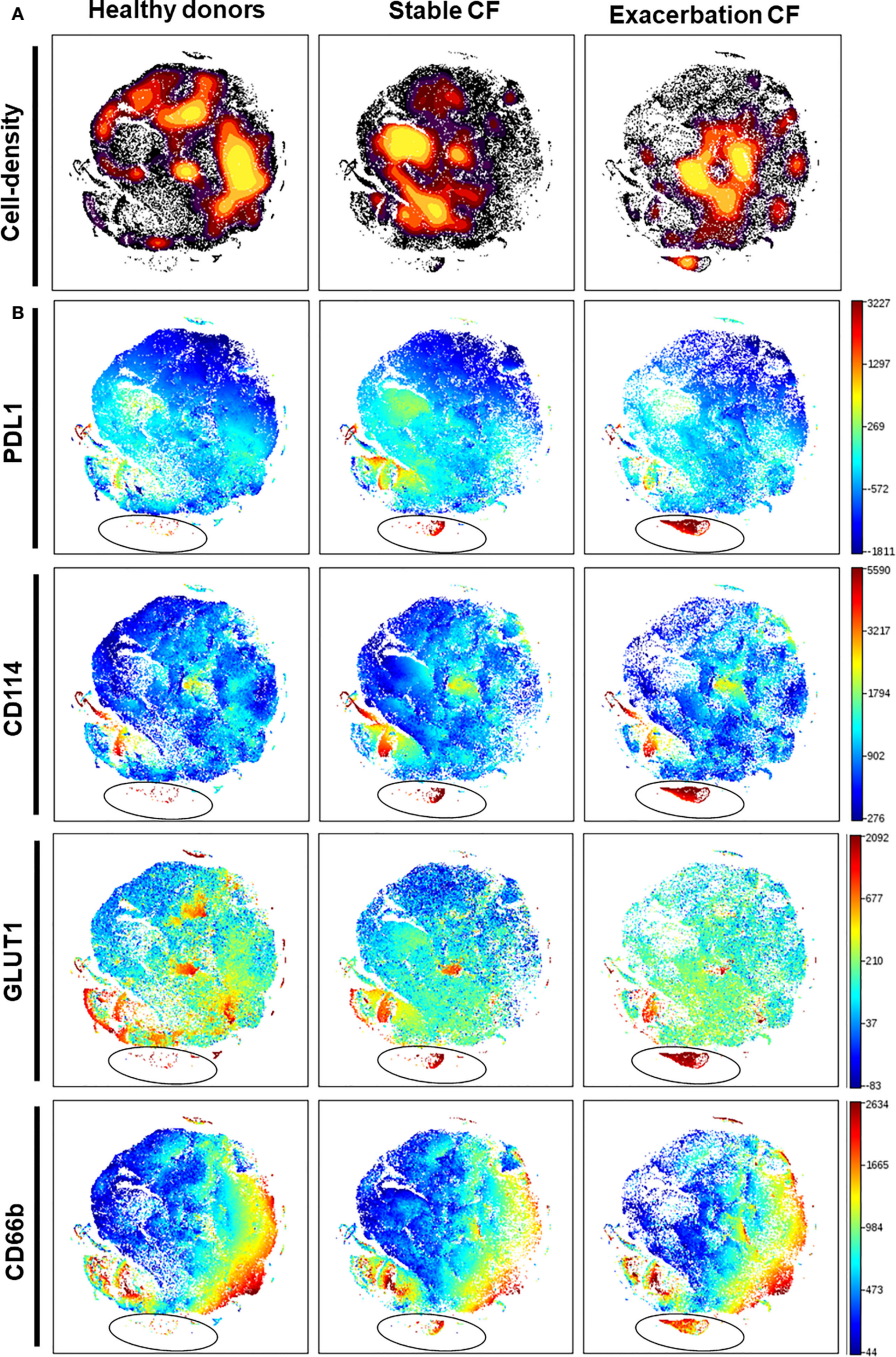
Identification of a CD114high/PD-L1high neutrophil subset in stable and exacerbation CF patients. An unsupervised analysis was performed, using the dimensional reduction algorithm visual t-Distributed Stochastic Neighbor Embedding (t-viSNE) on 10 000 neutrophils from each individual: healthy donors (n = 20), stable CF patients (n=16) and CF patients during exacerbation (n=6). Cells were clustered along t-SNE-1 and t-SNE-2 according to per-cell expression of CD16, CD62L, CD10, CD33, PD-L1, LOX1, GLUT1, CD11b, CD66b and CD114. **(A)** Plots representing cell density. **(B)** Plots representing expression of PD-L1, CD114, GLUT1 and CD66b are expressed with a rainbow heat scale for median fluorescence intensity. This unsupervised analysis identified a novel PD-L1high/CD114high neutrophil subset present in stable CF patients and further extended in exacerbation CF patients (circled on the PD-L1 plots).

**Figure 6:**
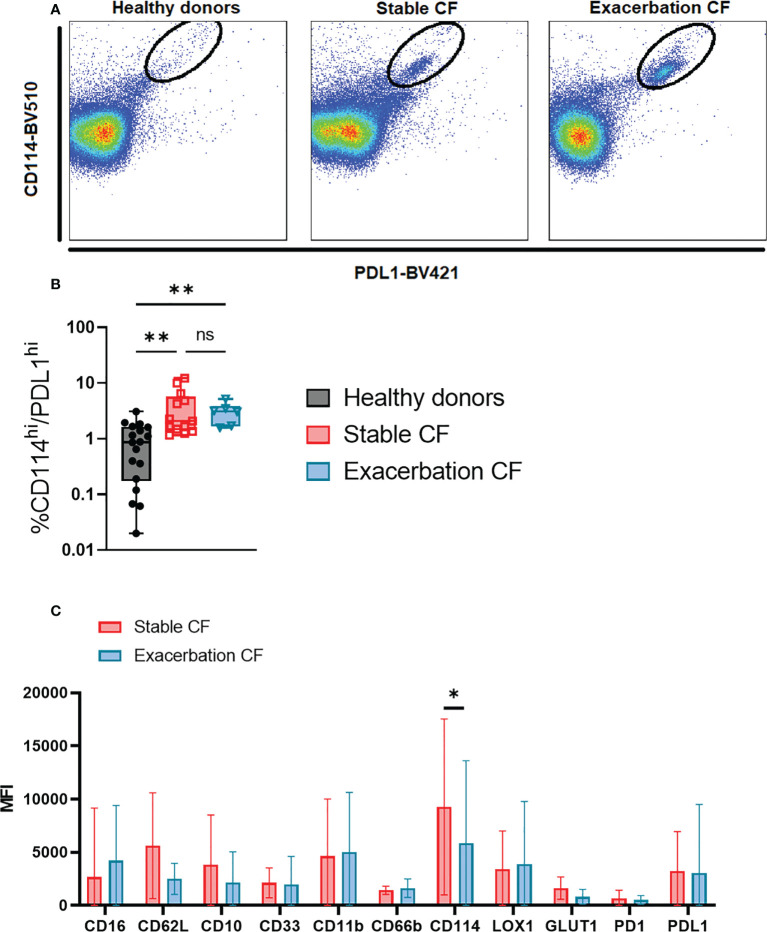
Phenotypic characterization of the identified PD-L1high/CD114 neutrophil subset in stable and exacerbation CF patients **(A)** Dot plots of concatenated files from each group, healthy donors (left panel, n=20), stable CF patients (middle panel, n=16) and CF patients during exacerbation (right panel, n=6) show the PD-L1high/CD114high subset. **(B)** Proportion of PD-L1high/CD114high subset within total blood neutrophils in healthy donors (n = 20), stable CF patients (n=16) and CF patients during exacerbation (n = 6) (Kruskal-Wallis with Dunn’s multiple comparison test, non significant (ns), **p < 0.01. **(C)** Comparison of PD-L1high/CD114high subset phenotype between stable and CF patients during exacerbation. Data are presented as median fluorescence intensity (MFI) ± interquartile range (Mann Whitney U test, *p < 0.05).

Whole blood neutrophils were analyzed by using t-viSNE. This strategy allows us to define an imprint for each sample group where each neutrophil is represented on a two-dimension map. We first identified on the cell-density maps different nodes demonstrating the presence of different neutrophil clusters in the three groups (**Figure 5A**). The non-supervised analysis of each marker identified a distinct neutrophil subset in the CF stable and exacerbation groups characterized by a high CD114 and PD-L1 expression (**Figures 5**, **6A, B**). This subset seemed to be particularly activated (CD66b^high^) and displayed increased metabolic activity evidenced by the marker GLUT1 (**Figure 5B**). When comparing this subset in stable and exacerbation CF patients (**Figure 6C**), we identified a lower CD114 expression in CF exacerbation group.

## Discussion

CF pathophysiology combines mucus abnormalities with chronic airway infection and a destructive neutrophil-dominated airway inflammation. Whilst several previous studies focused on the phenotype of airway neutrophils, we herein examined whether circulating neutrophil heterogeneity could occur in clinically stable condition and/or during a pulmonary exacerbation due to an infection and/or inflammation burden.

In the present study, we identified an increase in the CD16^hi^/CD62L^low^ subset, and the presence of a PD-L1^high^/CD114^high^ subset in clinically stable CF adults. During CF exacerbations, neutrophils displayed an increase in CD11b expression, and the CD16^hi^/CD62L^low^ and CD16^hi^/CD62L^low^ subsets increased. The identification of these neutrophil subsets in CF may help to quantify this chronic inflammatory state and help to diagnose exacerbations. Furthermore, this study contributes to identify specific neutrophil subtypes that may be targeted by future therapies to increase their antibacterial potential and limit their immune-suppressive capacities.

Neutrophil heterogeneity is a relevant topic in CF, although the debate on whether the different subsets of neutrophils are pre-existing bone marrow-derived populations of neutrophils or differentially activated neutrophils is not resolved (32). In this research, we used multiple approaches to study circulating neutrophil subpopulations in CF patients either in a stable clinical condition or undergoing pulmonary exacerbation. To our knowledge, this is the first study to investigate neutrophil phenotypes in CF with such a large panel of markers.

Regarding our patients sample, some limitations should be mentioned. The majority of patients were male, although we did not find any difference in the neutrophil phenotype of males and females. Second, it should be noted that eight patients were receiving anti-IL5 or anti-IL5R therapy for associated asthma. These drugs, targeted against eosinophilic inflammation, have no known effect on the neutrophil lineage. However some studies have suggested that the IL-5 receptor is expressed by circulating and airway neutrophils under inflammatory conditions in mice and humans (37). In our study, no differences were observed in any of the analyses performed between patients receiving such drugs compared to those not receiving them. The effect of anti-IL5 and IL5-R on the neutrophil phenotype is not conclusive in this study.

At first, our marker-by-marker analysis did not find a significant difference in the membrane phenotype of whole blood neutrophils between healthy donors and stable CF patients, whereas a significant increase in CD11b was observed in CF patients during exacerbations.

Secondly, using the double labelling of CD16/CD62L originally described by the Koenderman group (35), we showed that the major CD16^high^/CD62L^high^ subpopulation in healthy donors, was decreased in CF patients both when clinically stable and during exacerbations. Consequently, an increase in the CD16^high^/CD62L^low^ subset with a very mature phenotype, was observed in all CF patients. In contrast, the CD16^low^/CD62L^high^ subset with an immature and less activated phenotype was significantly increased only during CF exacerbations. This latter observation is in keeping with previous studies suggesting that this neutrophil subpopulation is present only in the first days after the onset of inflammation (36). Conversely, the CD16^high^/CD62L^low^ subpopulation persists for several days, and its significant increase in CF patients in a stable condition is consistent with a chronic inflammatory state in this disease.

To further document the phenotype of circulating neutrophils in CF, we performed an unsupervised analysis using a dimensional reduction tool thereby allowing to visualize the phenotype of every neutrophil with every marker. We identified a subpopulation that is hardly detectable in neutrophils from healthy donors and that is present in CF patients with a PD-L1^high^/CD114^high^ phenotype. PD-L1 has been described as marker of immunosuppressive neutrophils in sepsis (13, 38). PD-L1 expression on neutrophils is associated with injury-induced infection susceptibility (39) and promotes lung injury in murine sepsis model (40). Interestingly, PD-L1 has been shown to be expressed bimodally on CF airway neutrophils and was potentially involved in the modulation of the lung immune response (41). Interestingly, gamma interferon could upregulate the expression of PD-L1 on human neutrophils which could in turn acquire the capacity to suppress T cell proliferation (42). In the latter study, PD-L1 expression was restricted to the CD16^high^/CD62L^low^.

Notably, this specific PD-L1^high^/CD114^high^ subset is already present in stable CF patients and is further expanded during exacerbations. This is consistent with the high expression of CD114, the G-CSF receptor which is upregulated upon infection (43). Interestingly, the increased expression of GLUT-1, the receptor for glucose is consistent with the notion that neutrophils from CF patients have a metabolic reprogramming (44).

The proportion of LDN was not different in stable CF patients compared to healthy controls but was increased during CF exacerbations. These results confirm previous observation (45) suggesting that circulating LDN are not a major component of the CF pathophysiology, besides an acute exacerbation state. Furthermore, analysis of the phenotype of LDN in CF patients shows that their immature phenotype which was similar to those of healthy donor LDN might be endowed with immunosuppressive T cell properties (10). Indeed, it has been well described that within an heterogeneous population of CD66-positive neutrophils (either LDN or NDN) CD10 can be used to discriminate mature from immature neutrophils populations in G-CSF-treated donors or in patients with cancer or in systemic lupus erythematosus (46). In CF patients, we failed to observe any significant modulation of CD10 expression on whole neutrophils (or in the percentage of neutrophils expressing CD10). In contrast, a significant decrease in CD10 expression was observed in the LDN fraction in CF patients and in healthy donors confirming that these cells are immature. Apart of CD10, LDN from CF patients and from heathy donors show a significant decrease in the expression of CD16, CD62L and PD1 and a significant increase in the expression of CD33, CD66b, CD114 and LOX-1. The increased LDN percentage during infectious episodes leading to pulmonary exacerbation might affect the immune response. Interestingly, it has been previously proposed that *Pseudomonas aeruginosa* airway infection could modulates neutrophils by favoring the generation of myeloid-derived suppressor cells that expressed CD33, CD66b, LOX1 and PD-L1 (47). Other studies have been also focused on the influence of the airway environment on the induction of such phenotypes, and in particular the role of *Pseudomonas aeruginosa*, well-known to play a central role in the pathophysiology of CF (48, 49).

Most of the studies describing neutrophils in CF patients have focused on airway neutrophils, defining the GRIM phenotype (Granule-Releasing, Immunoregulatory, Metabolic attributes) (50), which is characterized by a higher primary degranulation, immunosuppressive abilities and a different metabolism, giving it resistance to apoptosis. Airway neutrophils, which have undergone trans-endothelial and then tissue migration, are then exposed to the pathological conditions of CF airways (hypoxia, influx of other immune cells, bacteria, fungi and others), which changes their phenotype (51).

Although CF micro-environment is obviously an important element to program neutrophils, endogenous neutrophil disturbances due to cystic fibrosis transmembrane conductance regulator (CFTR) defect remain to be studied. Indeed, it is now well-admitted that neutrophils express CFTR (52, 53).

From our present work we can conclude that there is a heterogeneity of neutrophils in CF which is different from those observed in healthy donors. What does this bring to our clinical practice?

First, the concept of exacerbation is difficult to define in CF, since the airway of these patients are colonized by bacteria and fungi, sometimes referred to as “chronic bronchial infection”. Because it is usually assumed that the infection is causing the exacerbation, aggressive antibiotic treatment is used to dampen these episodes. These drugs, sometimes administered intravenously and often for at least two weeks, have significant side effects, and their repeated use has led to the development of multi-resistant strains. However, it is likely that these exacerbations are amplified by the explosive neutrophil inflammation, driven by specific subsets of circulating neutrophils and on which the antibiotic treatment does not have a direct effect.

Furthermore, our long-term goal is to identify neutrophil subsets at different clinical stages of the disease and in response to CFTR modulators. These drugs are small molecules capable of correcting the defective protein and have greatly improved the management of CF patients. Treatment with Elexacaftor-Tezacaftor-Ivacaftor triple combination therapy (54), for which up to 85% of patients with CF are eligible results in large improvement in symptoms, respiratory function, and epithelial ion transport, including in patients with advanced lung disease (55). It appears logical to ask the question of the evolution of the phenotype of circulating neutrophils under the effect of these modulators. Characterization of such neutrophil subsets would require further studies, for example sorting the different subpopulations and investigate their function and proteomic profile. This would provide a signature of neutrophil phenotype and reprogramming as we have shown in patients with vasculitis (56). A current study performed in our laboratory on the effect of the Elexacaftor-Tezacaftor-Ivacaftor triple combination therapy on neutrophil phenotypes will bring some additional clues on the possibility to modulate or not neutrophils reprogramming in CF.

Finally, the need for alternative anti-inflammatory therapies is reinforced by observations that managing long term inflammation still remains a challenge despite implementation of CFTR modulator therapies. The neutrophil remains a target of choice to achieve this goal. Acting on neutrophil recruitment by aiming at the interleukin-8/CXCR2 pathway is also a very well-studied pathway (57). Target neutrophil survival pathways to promote apoptosis of a specific subset to favor the resolution of inflammation could be another option (26). Consistent with this notion, roscovitine has shown promising results in CF (58, 59).

The present work is of great importance since it shows that specific neutrophil subsets persist when a patient is considered as clinically stable therefore highlighting the endogenous a persistent neutrophil reprogramming that has to be corrected.

## Data availability statement

The raw data supporting the conclusions of this article will be made available by the authors, without undue reservation.

## Ethics statement

The studies involving human participants were reviewed and approved by CPP Ile de France XI (19005–2137-18.11.26. 50423). The patients/participants provided their written informed consent to participate in this study.

## Author contributions

All named authors meet the general criteria for authorship of this manuscript and have given final approval for publication. CM, TD, MA, SM, VK, FP, ML, JS, P-RB and VW-S designed experiments and analyzed data. MA, SM and VK performed experiments. CM, MA, SM, VK, JS, P-RB and VW-S contributed with critical reagents/tools/clinical samples. TD, CM, ML, P-RB and VW-S wrote the manuscript. All authors contributed to the article and approved the submitted version.

## Funding

This study was supported by grants from Vaincre la Mucoviscidose (RC20180502225 to VWS); from ABCF-Proteine (to VW-S); from Fondation pour la Recherche Médicale FRM (EQU202003010155 to VW-S); from the Investissements d’Avenir programme ANR-11-IDEX-0005-02, Sorbonne Paris Cite, LabEx INFLAMEX (to VW-S); from IDEX UP AAP EMERGENCE 2022 (RM27J21IDXC9) (to P-RB); from Fondation “Sauvez la Vie” (to P-RB); from La Fondation du Souffle, Master 2 fellowship (to TD).

## Acknowledgments

We are grateful for the excellent technical assistance provided by the CYBIO platform at the Institut Cochin and for the collaborative work with the team of the Etablissement Français du Sang for providing us blood from healthy donors.

## Conflict of interest

The authors declare that the research was conducted in the absence of any commercial or financial relationships that could be construed as a potential conflict of interest.

## Publisher’s note

All claims expressed in this article are solely those of the authors and do not necessarily represent those of their affiliated organizations, or those of the publisher, the editors and the reviewers. Any product that may be evaluated in this article, or claim that may be made by its manufacturer, is not guaranteed or endorsed by the publisher.
